# Chronic Adhesive Perimetritis

**Published:** 1884-09

**Authors:** James H. Etheridge

**Affiliations:** Professor of Materia Medica and Medical Jurisprudence, Rush Medical College. Gynecologist, Central Free Dispensary, Chicago, Ill.


THE CHICAGO

### Medical Journal and Examiner.

Vol. XLIX. SEPTEMBER, 1884. No. 3.

### Original Communications.

### Article I.

Chronic Adhesive Perimetritis. *By* James H. Etheridge.

*Professor of Materia Medica and Medical Jurisprudence, Rush Medical College. Gynecologist, Central Free Dispensary, Chicago, Ill.*

[*Read before the Chicago Gynecological Society, November*, 1883.]

*Definition and Nature.—Perimetritis* is inflammation of the pelvic peritoneum. This term was first proposed by Professor Virchow in 1862. He bases this name on its analogy with pericarditis.

M. Bernutz names this inflammation "*Pelvi-Peritonite.*" Scanzoni calls it "*Pelveo-Peritonœitis.*" Courty, "*Inflammation Peri-uterine.*"

Perimetritis is rarely idiopathic; it is nearly always symptomatic. It is possible that it may arise from direct injury, but in such cases it is as justifiable to suppose that some other organ—as the bladder, ovary, uterus, bowel or fallopian tube—

was the *first* to become inflamed, and the peritoneum *afterwards* became inflamed, as to contend that the peritoneum was the first to take on inflammatory action.

In nearly all cases of perimetritis the inflammation arises from an irritation applied to the peritoneum from the condition of the pelvic organs incident to menstruation, or from causes presented by the condition of the pelvic organs in the course of their diseases or abnormal conditions, which will be presently considered. It is never seen after the menopausis.

*Frequency.*—In a large gynecological practice one is surprised to meet with so many cases of perimetritis. In the virgin and in the child-bearing woman we find it exceedingly frequent. The one means of examination—*conjoined mainpulation*—almost at once reveals its presence. By touch we soon exclude cellulitis and extraneous pelvic growths, and obtain the tenderness and difficulty of movement of the uterus which plainly indicate adhesions. Speculum examinations also reveal, especially in virgins and women who have not borne children nor had a blenorrhagia, the peculiar vascular changes which clearly indicate impaired circulation in the uterus. Winckel states that he found perimetritic adhesions in more than 33% in all autopsies of women. Courty affirms that about one-third of uterine diseases is perimetritis. Aran found pelvic peritoneal adhesions and evidences of previous inflammatory action in 55% of women examined post-mortem. He states that "adhesions were twice as common in women who had borne children as in women who had not." Heitzmann affirms, in 1883, that fully three-fourths of Professor Bandl's clinic present evidences of past or present perimetritis.

Duncan states that "In women not recently confined, diseases of the genital organs and accidents, surgical or other, lead to adhesive perimetritis more frequently than to pelvic inflammation of any other seat or nature."

The remarkable frequency of this condition certainly demands careful study. It is a matter of great surprise to me that so little is written upon this topic.

*Anatomical Considerations.*—It is of the utmost importance that our knowledge of the peritoneal relations be exact. Let us review them briefly:

The pelvic portion of the peritoneum presents the appearance, when viewed from above, of having been pushed up from beneath, from the bottom of the pelvis, by the uterus, its appendages and the bladder. Upon sawing perpendicularly through the frozen female pelvis from the symphsis pubis to the middle of the sacrum, and removing the abdominal viscera in order to dissect with ease, it will be found everywhere that the pelvic organs push up the peritoneum, the latter is protected from absolute contact with them by a film of connective tissue, thin in all places and very thin in a few of them. This connective tissue is called a "muscular platysma" by Savage, and furnishes, he says, for the most part, the surgical interest of the pelvic peritoneum. Its peculiar structure can be studied in the reduplications of the peritoneum.

The impression cannot be escaped when one considers the ubiquity of the peritoneum in the upper part of the pelvis, folded around every one of its organs to a greater or less extent, and projected between them in the shape of folds and ligaments, that Nature intended to crowd as much of this organ into the pelvic cavity as could be well accommodated there.

*The Broad Ligaments.*—These ligaments are composed of the two layers of peritoneum passing from the anterior and posterior surfaces of the uterus outwards and backwards to seek attachment to the pelvic wall just anterior to the sacro-iliac synchondrosis, the layers separating at the points of their attachment and at their bases. Containing between their laminæ the fallopian tubes, ovaries, blood-vessels, lymphatics, connective tissue and unstriped muscular fiber, they present the beginnings of many attacks of pelvic peritonitis—consequently their anatomy and relations cannot be too thoroughly known.

Their position varies according to the location of the uterus. Normally they lie obliquely, their posterior surfaces looking upwards and backwards, and their anterior surfaces looking downwards and forwards. In pregnancy they are drawn up and lie almost vertically. In backward displacements of the fundus they are twisted longitudinally coincidentally with the degree of the displacement. It will be found in many patients that the greater the retroversion or the retroflexion the stronger are the objective symptoms of chronic perimetritis, doubtless facilitated in its access primarily by the uterine displacement.

The broad ligaments and the uterus divide the pelvic peritoneal cavity into two unequal divisions. The anterior cavity contains the bladder only, and is much the smaller of the two. The posterior lamina of the broad ligaments extends farther downwards than the anterior, especially in the middle, covering the upper and posterior part of the vagina. The anterior lamina dips down only to the level of the internal os uteri, and is then reflected on to the bladder.

Viewed from above, the broad ligaments present the appearance of having three minor folds embracing from before backwards the round ligament, fallopian tube and ovary respectively. These folds occupy the posterior fossa of the peritoneal cavity. Inflammation of the pelvic peritoneum commences more often in the posterior than in the anterior fossa, and anatomical relations explain the reason therefor. When the fundus uteri is turned backwards its malposition constitutes one of the etiological factors of perimetritis. Fluid escaping from the fallopian tubes, whether it be purulent, gonorrhœal, sanguineous or medicated, will be deposited in the posterior pelvic peritoneal fassa and produce perimetritis.

*The Utero-Sacral Ligaments, or Folds of Douglas.*—These ligaments are crescentic layers of peritoneum reflected from the broad ligaments above them, and muscular platysma containing muscular fibres from the vagina and uterine cortex (Savage), which pass from the lower, lateral part of the uterus, opposite the os internum, at about the insertion of the vagina, outwards and backwards for insertion to the inside of the sacro-iliac symphysis at the third sacral vertebra, and, often, above as high as the promontory, or the anterior and lateral part of the last lumbar vertebra. (Huguier). They form the upper and lateral boundaries of the fossa of Douglas, which communicates with the peritoneal cavity through a lumen between these ligaments. In this lumen lies the empty large intestine. In the normal state they act as one of the factors of the support of the uterus *in situ naturale*, contributing to prevent downward and forward displacements of the cervix.

*The Fossa of Douglas.*—This fossa is a pouch of pelvic peritoneum lying between the uterus and vagina anteriorly, and the rectum and sacrum posteriorly. It is, anatomically, the lowest projection of the peritoneum. This cavity is of the gravest importance practically. It can easily be explored through the vagina and rectum. The utero-sacral ligaments bound it above, and, below it, usually extends to the vaginal fornix. Exceptionally it extends to the floor of the perineum. (Pirogoff).

Unrestricted by adhesions, this cavity may attain dimensions equal to the cavity of the pelvis, exclusive of the thickness of the rectum, vagina and bladder. Into this cavity various organs—as the small intestine, the fundus uteri, or the ovary, or even an extra-uterine fœtation—may protrude. Serous, sanguineous or purulent fluids are frequently found in it. And lastly, the fibrinous adhesions of perimetritis are often found in this cavity. Its dependency makes it especially liable to take on inflammatory action from the invasion of exciting causes from above.

Its distensibility and capacity for dislocation are astonishing. It has repeatedly been found protruding from the vulva, pushing the posterior vaginal wall before it, and, in other cases, from the anus, pushing the anterior wall of the rectum before it. In one patient I saw the fundus uteri retroverted and retroflexed till it rested on the perineum, and it could be readily grasped by the thumb and finger introduced respectively into the vagina and rectum.

The lowest part of the fossa lies distinctly to the left of the uterus. It is stated that the fossa is of equal depth on both sides, chiefly in old women and in presumed pathological processes. (Barnes). Very often this space can be found empty, and the examining finger can be thrust into it, inverting the peritoneum and feeling the utero-sacral ligaments right and left, presenting various degrees of thickness, tautness and tenderness. In such an examination, best conducted in the anæsthetic state, the finger displaces whatever is in front of it, whether it be the uterus, an ovary, a fold of small intestine, or an extra-uterine fœtation.

The careful differential examination of this space yields, very often, the most valuable information to the gynecologist. When replete it is always swelled and sometimes descended. When occupied by a solid organ its outline through the walls of the fossa are easily and sharply defined. When filled with hardened lymph, as in the case of *colica scortorum*, its outline is hard, smooth and uniform, and the space is descended partially. When filled with fluid, of which there is a large accumulation above—as blood, serum, or pus—its greatest descent and distention are observed. Often in such cases it fills the whole vagina—nay, even, at times, the whole pelvic basin, so that finding the cervix is difficult, and in some instances impossible. Cutting into the Douglas fossa, when in its natural relations, without doing more than is intended, is only too often impossible; whereas, cutting into it when largely distended is a very easy matter. Indeed, thrusting the finger into it upon making a digital examination occurred once to my friend Prof. Parkes, and it was immediately followed by an enormous discharge of pus. A case of hydatids of this fossa is described, wherein an abscess formed and protruded beyond the vulva, forming a tumor larger than an adult fœtal head. (Duncan). Freund reported a case of great procidentia, where the fossa of Douglas descended unchanged, carrying with it the rectum to the lowest part of the prolapsed mass.

PATHOLOGY.—Three or four forms of inflammatory changes in the pelvic peritoneum are described by authors:

1st. *The Adhesive* form, the commonest of all forms of inflammation of the pelvic organs;

2d. *The Suppurative* form, the most important because of its frequently fatal termination; and

3d. *The Serous* form, the rarest of all forms of pelvic peritoneal inflammation.

To the foregoing subdivision, some writers add:

4th. *The Sero-Adhesive* or *Sero-Encysted* form, a combination of the first and third forms.

I desire to call attention to the chronic stage of the adhesive form of perimetritis. We see perimetritis very often in its chronic stage, and but rarely in its acute stage.

When we reflect that all inflammation is *active*, it would almost seem that *chronic* inflammation is paradoxical. One writer goes so far as to declare that there is, strictly speaking, no such process as *chronic inflammation;* while, upon the other hand, some inflammations are said to be "*chronic*" from their inception. (Courty.) However, I wish to include in this article those cases which have passed beyond the first or acute stage, as so understood, attended by constitutional disturbance of a pyrexial character.

The etiology of perimetritis will be better understood if we bear in mind that serous membranes are simply modifications of the unformed connective tissue of the body which may be regarded as a "continuous whole in which the nervous and muscular fibres, bone and cartilage, epithelial tissues, etc., are embedded. * * * * The connective tissue of the serous membranes is uninterruptedly continuous with the interstitial connective tissue of those organs which they invest. The inference from this is plain: that the serous membranes may and do, in fact, take part in all the morbid changes to which the interstitial connective tissue of those organs is liable." Bearing this fact in mind will enable us to understand how an inflammation of any of the pelvic organs may eventuate in a pelvic peritonitis. (Rindfleisch.)

The course of the inflammatory process is determined in accordance with the respective intensity and quality of the great variety of irritants producing it. The process observed in the acute stage is a reddening of the peritoneal surface, which is the capillary engorgement preceding the migration of the colorless blood corpuscles, fibrin and serum. From this point the further histological metamorphosis tends to one of three directions, viz.:

1st. Towards *Resolution*—*i. e.*, to the absorption of the exudation and a return of the peritoneum to the condition in which it was before the inflammation set in;

2d. Towards *Organization*, wherein the well-known "bands of adhesion" are found; and,

3d. Towards *Suppuration.*

Cases terminating in resolution may be said not to pass into the chronic stage at all, because the steps towards the pathological acme and back again to health are so rapid that none of the appearances of slowness of changes so commonly associated with the chronic form of inflammation are herein seen. It may be well to remark that very many cases of this form of perimetritis occur after miscarriages and labor at full term, when the patients are confined to the bed and are thus subjected to the best of all therapeutic measures for this malady, viz., *rest.* Cases terminating in speedy resolution without doubt have causes that are comparatively mild in their force.

Cases terminating in organization are usually insufficiently considered by writers. After the *fibrinous* exudation has ceased its active movement it becomes physiologically equivalent to embryonic tissue, developing (1) the round into the spindle-shaped cells, whose processes touch each other and then become fused together, and, later, (2) the process of vascularization. The *serous* exudation either remains free in the pelvic cavity, or becomes encysted by the formation of false membranes, or is absorbed. After the fibrinous exudation has become organized the pelvic organs will become anchylosed to a degree *pari passu* with the amount of the exudate. By digital examination the part of the pelvis above the vagina will be found as "hard as a deal board," the normally movable organs above being matted together and quite indistinguishable by conjoined palpation. This stage constitutes a point of departure for very many cases wherein the organized material slowly disappears by absorption, stopping at almost any point from the slowest possible advance up to its complete disappearance. The rarest of all forms of perimetritis, viz., the serous form, is developed right here where the plastic exudate has nearly disappeared, and the encysted collections of serum remain, above, unaffected. Where neither exudate nor serum is wholly absorbed we have the *sero-adhesive* form of perimetritis which some authors have seen fit to mention especially. Two explanations for the increase of the serous fluid exist: One is, the increased peritoneal exhalation from retarded venous circulation from cardiac, hepatic or renal disease; and the other is, diminished absorptive capacity of the peritoneum. When a portion of peritoneum becomes inflamed its absorbing power is decreased, and where sealed spaces formed by inflammatory agglutinations of the pseudo-ligaments of perimetritis are found, serous accumulations will form of varying size. In this way serous cysts arise around the uterus. They are very commonly found in the fossa of Douglas. They vary infinitely in size, from that of a mustard seed up to that of a fœtal head. Now and then they attain to a large size, and they have been diagnosticated and operated on as ovarian cysts. M. Huguier, in 1847, suggested that the spontaneous cures of alleged ovarian cysts, after tapping, were really cures of cysts about the uterus and not cysts of the ovary.

Many cysts of the broad ligament have their origin in adhesive perimetritis. It is well known that they are usually not large, and are monolocular. When we consider the fact that sealed spaces in chronic perimetritis are at first small and may include in their extent a small surface of peritoneum capable of exuding the fluid of these cysts, we readily understand why they are monolocular and why the increase in the amount of fluid will cause an increase in the capacity of the cyst walls till pressure from within or extraneous influences cause a cessation of serous exudation into the cyst, and thus allow it to remain the same in size indefinitely. I have in my possession, from one of my oöphorectomy cases, a broad ligament cyst of this kind. The cyst walls are composed of exudation covering the broad ligament anteriorly, and they had a capacity of about two ounces.

Another fact of pathological interest is the co-existence of serous and suppurative perimetritis in the same patient. A sealed chamber of serum may exist in the same pelvis wherein a suppurative accumulation exists. I have no means of knowing whether they usually develop in the course of the same inflammatory attack or from separate attacks. It can be readily seen that it is possible for them to develop together or separately. Last month, for Dr. Dodge, of this city, I opened an abscess, as I supposed it to be, by cutting into the bulging fossa of Douglas. The patient had been ill in bed for six weeks with the usual clinical history of perimetritic abscess. I was astonished to see a great flow of serous fluid—about twenty ounces, I should say. We left the patient, protesting our chagrin after such an apparently clear clinical case of suppurative inflammation. The next morning we were still more astonished to learn that during the night the abscess itself had broken spontaneously into the rectum and discharged very freely. In this patient suppurative and serous perimetritis co-existed. She had been complaining several months previously, and it is highly possible that the serous accumulation was of a more ancient origin than was the purulent accumulation. The sudden supervention of the pains, rigors and fever six weeks prior to operating, clearly indicated the history of the suppurative development.

The *location* of the exudation at once reveals the fixation ot the pelvic organs. In severe cases the uterus, tubes, ovaries, bladder and rectum are seen to be held in an unyielding grip. The fossa of Douglas is more or less bulging. Any previously existing version or flexion of the uterus is found to be more pronounced. The pressure on the pelvic nervous supply often forms a conspicuous feature of the disorder. The same pressure also changes the physiological functions of menstruation and conception in the majority of cases. Often the exudations are found above the pelvic inlet and in the iliac fossæ.

The *amount* of the exudation, as shown by the extent of the adhesion, is exceedingly variable. The fixation of the pelvic organs ranges all the way from the slightest stiffness of movement of them by conjoined manipulation up to their complete immobility. The adhesions are found most commonly about the tubes and ovaries when very small in extent. They are seen of all kinds of density, but frequently as "bands of length and thickness such as only imperfectly to impede motion—evidence in themselves of a gradually progressing change from close adhesion to complete loosening." (Duncan). Such small adhesions about the tubes and ovaries are doubtless the last remnants of previous perimetritis.

The length of time of the presence of the adhesions varies almost indefinitely. Very many patients present cases of pelvic peritonitis, with *partial* anchylosis of the pelvic organs, after a menstruation, which almost completely disappear before the next menstruation. Again, we will find cases of *complete* anchylosis of the pelvic organs, resulting, *e. g.*, from miscarriages, which will completely disappear in from four to eight or twelve weeks. Then, again, we meet with cases wherein there is *permanent* anchylosis, *i. e.*, where it persists for years.

Generally speaking, the ultimate tendency of pelvic inflammatory exudate is towards its spontaneous absorption. Many cases are "cured" by a pregnancy, wherein they are stretched into bands so fine that their nutrition is practically cut off, and they remain after parturition as shrivelled up, elongated attenuations of tissue, devoid of any power to fix the uterus. Herein we have the explanation of the improvement in general health so often met with in women who are "much healthier" after a confinement than they were before it.

If we accept the usual movements of the pelvic organs incident to the movements of the body, of respiration, of coughing and of sneezing, as the primary cause of irritation leading to absorption of the exudate, we can understand easily how that portion of the adhesions about the ovaries and tubes should persist after all the others had disappeared, because of the diminished motion of those organs.

The variation of dislocation of the tubes and ovaries cannot be even approximately described. Sometimes the ovary is distorted and fastened by a few membranes; then, again, it is so imbedded in a mass of bands and membranes that it is found only with the greatest difficulty. "Thus the left ovary may lie in a curvature of the sigmoid flexure and the right may be adherent to the vermiform process in a mass of exudation." Sometimes the uterus becomes imbedded in false membranes and undergoes premature atrophy. The ovary may undergo the same process. The fallopian tubes may become compressed and even detached from the uterus. Their ends are often stenosed or sealed hermetically, and their lining membrane may go on pouring out purulent or serous fluid according to their condition previous to the occlusion, and thus the tubes become enlarged to an enormous extent, presenting so-called cases of pyo and hydro-salpinx. Where the contained fluid is blood—the fluid most rarely found in fallopian occlusions—the case is called haemato-salpinx. Peaslee describes a case of fallopian distension wherein eighteen pounds of fluid were contained. Adhesions between contiguous coils of intestine may lead to attacks of constipation of a most rebellious character, alternating with diarrhœa and colic. Active cell proliferation in the connective tissue of the outer muscular uterine fibres leads to the development of an inelastic capsule over the uterine parenchyma, which becomes a prolific cause of abortions where pelvic peritonitis has previously existed. A similar condition existing around the ovary prevents the bursting of a ripened follicle, and gives the first impulse to the development of an ovarian cyst in some cases, and leads to barrenness in all cases thus elaborated. A large proportion of displacements of the uterus is doubtless due to perimetritic exudations and adhesions, which, when unrelieved, lead to abortions and sterility. Occasionally the plastic exudate becomes vascularized and hyperæmieted to the point of causing hemorrhages by a repetition of inflammatory attacks, and we have then a new danger to deal with, viz.: *hematoceles.* This condition has been denominated *hæmorrhagiparous pachy-peritonitis.*

ETIOLOGY.—In considering the causes of perimetritis we must never lose sight of the fact that it is rarely, if ever, idiopathic. I have never seen a case that was not secondary to some previously induced pathological condition. By far the largest number of cases arise from uterine, tubal and ovarian disorders.

The following causes of pelvic peritonitis may now be considered in detail:

I. Menstrual disturbances.

II. Pregnancy terminating in abortion or full-term delivery.

III. Gonorrhœa.

IV. Traumatism.

V. Pressure.

VI. Parametritis.

I. The influence of cold upon the menstruating woman must be regarded as one of the most prolific causes of perimetritis. Every month the uterus and its adnexa are congested to a superlative degree, and it becomes an extremely easy matter, pathologically, to convert this normal into an inflammatory condition. The inflammation, once started, is limited in its ulterior progress only by conditions not now fully understood. As elsewhere stated in this paper, perimetritis may cause almost no inconvenience at the time of its progress, or it may result in a permanent invalidism, or even death. Between these two extremes we will find every grade of severity of pathological developments and symptomatology.

The etiology of adhesive perimetritis will undoubtedly, in the largest number of cases, be found in the partial or complete suppression of the menses from cold. The latter may be applied in a variety of ways, either by exposure, by getting wet in storms, by sitting on cold stone steps, immersing the hands in cold water, washing the genitalia in cold water, getting the feet cold, sleeping in damp sheets, eating cold ices, etc., etc. Occasionally it will be found that cold, between menstrual epochs, will produce perimetritis.

Any sudden diminution or suppression of the menses is very liable to produce perimetritis. Gradual diminution of the flow leading up to its disappearance is liable to result in this inflammation, Within the past year I saw a patient decreasingly menstruate for three consecutive months, and on the fourth she failed to flow at all, but developed a perimetritis instead. Incomplete menstruation from no apparent cause may be the cause. Occasionally, without known cause, the flow will cease and perimetritis will follow,—a condition which Tarnier has denominated "a puerperal fever occurring in the course of menstruation." Severe mental emotion will also cause a suppression of the menses, which may eventuate in perimetritic inflammation. Local treatment during menstruation may also produce this malady. The same may be said of excessive sexual intercourse during menstruation.

II. Bernutz presented ninety-nine cases of perimetritis, with their causes, and he attributed forty-eight cases—by far the largest number under any one etiological factor—to the puerperal condition. Puerperal perimetritis includes all cases arising in the puerperal condition, whether the pregnancy has terminated in full term or premature delivery. Whether the causation result from sepsis, or from an extension of the inflammatory condition from the uterus, it is not always easy to decide—in fact, it would be extremely hard to decide, in the majority of cases. Too early rising from confinements must be regarded as a common cause of perimetritis. Any indiscretions of the puerperal patient which would produce peritoneal inflammation would be comparatively harmless at any other time; hence the conclusion must be reached that puerperality is an important etiological factor in this disease. When the very large number of cases of perimetritis that exist is taken into consideration, I think that the parous condition will be placed second to menstrual condition as an etiological factor.

III. Gonorrhœal inflammation, extending along the endometrium into and through the fallopian tubes to the serous membrane, is communicated to that organ with unerring certainty. Nearly, if not quite, all women who have a blenorrhagia, sooner or later develop perimetritis. In this manner develop cases of *colica scortorum.* Bernutz denominates this condition—gonorrœhal perimetritis—"a veritable feminine orchitis." However, the resemblance of epididymitis to pelvic peritonitis was first enunciated by Rochoux in 1833.

IV. Violence, in its multiform applications, can produce this inflammation. Sounding, curetting, tents, stem pessaries, cutting operations in the uterus, the application of the solid nitrate of silver to the interior of the cervix, injections of fluid into the endometrium, venereal excesses and chancrous ulcerations on the cervix have produced pelvic peritonitis. Thomas includes, also, "blows, falls, injury during labor, and punctures" as kinds of violence producing this malady.

V. Pressure arising from an enlarged uterus, flexions and versions of the uterus, an enlarged or displaced ovary, chronic constipation, pessaries, an ovarian cyst, myomas, carcinomas and hematoceles may give rise to pelvic peritonitis.

VI. Parametritis is in most cases accompanied by perimetritis of various degrees of intensity. The former may, and undoubtedly often does, develop or run its course without the latter, in mild cases. Chronic parametritis of comparatively long standing, without an accompanying perimetritis, may become a cause of a renewal of an acute cellulitis, which in course will produce a severe perimetritis, eventuating even in suppuration. Within the past two months I have attended three such cases.

COURSE AND SYMPTOMS.—For convenience of description of the course and symptoms of pelvic peritonitis, we may divide cases into two classes, viz.: (1) *Mild cases* and (2) *Severe cases.*

1. *Mild* cases may run their course, unobserved at the time, at least, by the physician. They comprise by far the largest proportion of all cases of adhesive perimetritis. I think it is safe to assert that the vast majority of mild cases arise in the course of menstruations where *cold* is the etiological factor. The menstrual discharge is diminished, and is accompanied by pain in the hypogastric region in varying degrees of intensity. This pain is such a common symptom that, usually, domestic remedies only are used, such as rest, hot applications externally, and stimulating drinks. The pains soon cease, the menstrual period passes by in due time, and the patient resumes her usual habits. In the mildest cases all symptoms cease at this time, and, at most, are experienced at one or two subsequent menstrual epochs, or they do not recur again till a fresh cause is applied. In the severer of the mild cases the patient continues to suffer at each subsequent menstruation. In a small proportion of these severe cases the patients have the dysmenorrhœal sufferings every second month, the alternate months no suffering being experienced. If it be true that each ovary is active only at every second menstrual period, it is possible that the explanation of the pain supervening at alternate menstruations only lies in the fact that one ovary is involved in perimetritic adhesions, while the other is free from them.

Very commonly after the commencement of adhesive perimetritis the patient can recall at some one menstruation, she was dysmenorrhœic for an indefinite period only—*i.e.*, pain at menstruations is the only trouble complained of. Sooner or later—it may be one month and it may be one year or longer—she evinces symptoms of inflammatory changes in the uterus, or of uterine dislocation. These symptoms intensify till her general health suffers, and then it is that she seeks medical advice. This period varies anywhere from one month to years after the initial departure from health. In these cases it is most interesting to trace the steps of the malady from its inception to date. Only too frequently the defective memory of the patient prevents the tracing the anamnesis. Very many times the patient voluntarily states the time when the suppressed, painful menstruation occurred, and adds, "And since that time I have never been as well." Without painstaking in tracing out the history of these cases, gynecologists are only too often led to say that this malady will run its course unobserved. In many of the cases of perimetritis the only evidence of it ever adduced is found in the adhesions found post mortem.

2. *Severe cases.*—In this class of cases the symptoms are more pronounced. The pain is severe, the febrile movement decided, and the constitutional symptoms marked. A chill may or may not precede the other symptoms.

The physician who tries to make the initial symptoms tally with the ensemble of symptoms of general peritonitis will signally fail. The constitutional depression associated with the latter grave malady will be conspicuously absent.

The symptoms most commonly present in the acute stage are:

(*a*). Pain.

(*b*). Tenderness.

(*c*). Febrile movement.

Additionally, there are often:

(*d*). Nausea, vomiting and tympanites.

(*e*). Bladder and rectal irritability, and bearing down.

(*f*). Anxious facies and delirium.

I desire to speak especially of pain, only by way of amplification. It is found present in all possible degrees of difference. Many patients experience it but seldom, at long intervals, while others may be said never to be free from it, and between these two extremes are found all shades of its variations.

The most complaint of pain is made at the menstrual periods —in other words, dysmenorrhœa is a very common feature of perimetritis. *Per contra*, one will be surprised, whose attention has not especially been directed to this subject, to learn how many cases of dysmenorrhœa have chronic perimetritis for their cause.

Pains denominated "*neuralgic*" in character are an extremely common feature of this malady. They are very commonly called "enteralgia" and "ovaralgia," the supposed pathology being in the bowel and in the ovary. They are induced or exaggerated by various causes, notably by cold, fatigue and mental emotion. Patients harassed by such pains go from one doctor to another, unrelieved, till a fresh attack of inflammation, resulting possibly in abscess or in permanent invalidism follows. Times without number, almost, have I been told by patients that, when they are *chilled*, they will have pains in one or both groins, or in the entire hypogastric region, extending in many instances down the thighs, or around to the back, or up to the hypochondria, or, as one patient expressed it, "from her ribs to her knees." Again they will complain that, when fatigued, sharp, shooting pains pass through the lower abdomen, till rest or stimulation can restore their nervous balance. Occasionally mental depression will be followed by these pains. One patient informed me that moving suddenly, "any jarring, as jumping or being pulled around by another person," always produced a series of pelvic pains which were relieved only by opium or alcohol. Many patients are compelled to abandon singing, by the "abdominal method," because of the suffering induced thereby.

COURSE AND SYMPTOMS.—Later, some or many of the following symptoms will be noticed:

(*a.*) Sensitiveness, which continues for a long time and easily returns.

(*b.*) Abdominal pressure, from exertion or otherwise, causes pain.

(*c.*) Moving the uterus, often excessively tender.

(*d.*) Displacement of ovaries and tubes.

(*e.*) Sterility.

(*f.*) Menorrhagia.

(*g.*) Amenorrhœa.

(*h.*) A tumor, or a matting together of all the organs above the vaginal vault. Often arterial pulsations at its base are found. (Nonat.)

(*i.*) Uterine cervix movable, while the fundus is immovably fixed, "as though nailed to the sacrum." (Duncan.)

(*j.*) Douglas's fossa filled with adhesions often containing the fundus uteri, an ovary, or a coil of small intestine.

(*k.*) Irritability or neuralgia of the uterus.

(*l.*) Reflex paralysis.

(*m.*) An obstinate sciatica.

(*n.*) Extrauterine or tubal pregnancy.

(*o.*) Recurrent attacks of perimetritis.

(*p.*) Hematoceles from hemorrhagiparous peritonitis.

(*q.*) Hysteria; insanity.

(*r.*) Alternate constipation and diarrhœa.

*Diagnosis.*—Absolute correctness of diagnosis in all cases is impossible of attainment. Furthermore, the advantage to be gained from exactly diagnosticating the location of periuterine inflammations is often of no special value in treatment.

(*To be continued.*)

